# Supplementary material for: Content-rich biological network constructed by mining PubMed abstracts
Source: BMC Bioinformatics. 2004 Oct 8;5:147. doi: 10.1186/1471-2105-5-147 (PMC528731; doi:10.1186/1471-2105-5-147)
Supplement: Additional File 5 — The original Chilibot query results of the term "long-term potentiation (LTP)" and 22 other terms, limiting the latest references analyzed to the years 1990, 1995, 2000, and 2004. [file 1471-2105-5-147-S5.bz2 › chilibotAdditionalFile5/ltp2004/html/CAMKII_CAMKIV.html]

 


 **CAMKII** and **CAMKIV** 
  
Found 24 abstracts in PubMed,  **24 abstracts were retrieved and analyzed**.  


---

 Search Google  |
 PDF files only 
|  EDU domain only 

---

**Interactive relationship** (e.g. stimulation, inhibition, etc)

- The specific  **CaMKII**  inhibitor KN 62 50 microM had no effect on the  **CaMKIV**  activity and pancreatic enzyme secretion elicited by CCK 8, CCh and CCK OPE.  Ref: 10838169 Biochim Biophys Acta, 2000
- The results indicate that  **CaMKIV**  is much more potent than  **CaMKII**  in activating CREB in three different cell lines.  Ref: 7958915 Genes Dev, 1994
- Because both  **CaMKII**  and  **CaMKIV**  can phosphorylate CREB, we pursued further the mechanism by which  **CaMKII**  and  **CaMKIV**  differentially regulate CREB activity.  Ref: 7958915 Genes Dev, 1994
- Similar anti proliferative effects of KN 62 occurred in SK N SH human neuroblastoma cells, which express both  **CaMKII**  and  **CaMKIV** , and in K562 human chronic myelogenous leukemia cells, which express  **CaMKII**  but not  **CaMKIV** .  Ref: 8615909 Biochem Pharmacol, 1996

**Parallel relationship** (e.g. studied together, co-existance, homology, etc.)

- Immunoprecipitation and immunoblotting studies using a  **CaMKII**  or  **CaMKIV**  antibody showed that rat pancreatic acini expressed both  **CaMKII**  and  **CaMKIV** .  Ref: 10838169 Biochim Biophys Acta, 2000
- Calcineurin,  **CaMKII** , and  **CaMKIV**  activities increased at 2 minutes and peaked by 1.  Ref: 11073891 Circ Res, 2000
- These findings indicated that LIF activated  **CaMKII** ,  **CaMKIV** , and calcineurin through an increase in I Ca, L and calcium i.  Ref: 11073891 Circ Res, 2000
- The fairly high degree of the substrate specificity of this protein phosphatase suggests that the physiological significance of the phosphatase may be the regulation of the three multifunctional calcium calmodulin dependent protein kinases, CaMKI,  **CaMKII** , and  **CaMKIV** , which are the key enzymes in a calcium signaling system in the cell.  Ref: 9875237 Biochem Biophys Res Commun, 1998
- Although both  **CaMKII**  and  **CaMKIV**  activities converge in promoting survival, their actions on axon growth are markedly different Transfection of truncated  **CaMKII** ... into SGNs prevents axon outgrowth.  Ref: 12671991 J Neurosci Res, 2003
- The expression of both  **CaMKII**  and  **CaMKIV**  by SCLC cells, and the sensitivity of these cells to the anti proliferative effects of KN 62, suggest a role for CaM kinase in regulating SCLC proliferation.  Ref: 8615909 Biochem Pharmacol, 1996
- Examination of the subcellular distribution of transiently expressed  **CaMKIV**  and  **CaMKII**  reveals that only  **CaMKIV**  enters the nucleus.  Ref: 8065343 Mol Cell Biol, 1994
- ...  **CaMKIV**  had a small effect, and  **CaMKII**  had no effect.  Ref: 12193581 Endocrinology, 2002
- We also demonstrated that SCLC cells express both CaM kinase type II  **CaMKII**  and CaM kinase type IV  **CaMKIV** .  Ref: 8615909 Biochem Pharmacol, 1996
- While  **CaMKII**  has been implicated in learning and memory, the biological role of the other multifunctional CaM kinases, CaMKI and  **CaMKIV** , is largely unknown.  Ref: 10673339 Genomics, 2000
- Third, transfection of COOH terminal truncated lacking regulatory domain, constitutively active  **CaMKII**  or  **CaMKIV** ... full length CAMKs, promotes SGN survival in the absence of other trophic stimuli, indicating that CaMK activity is sufficient to promote survival.  Ref: 12671991 J Neurosci Res, 2003
- Although  **CaMKII**  and  **CaMKIV**  use autocamtide II as a substrate, peptide gamma is more efficiently phosphorylated by  **CaMKIV**  than by  **CaMKII** .  Ref: 10838169 Biochim Biophys Acta, 2000
- KN62 an inhibitor of  **CaMKII**  and  **CaMKIV**  attenuated c fos, brain natriuretic peptide, alpha skeletal actin, and atrial natriuretic peptide expression.  Ref: 11073891 Circ Res, 2000
- The aim of this study was to investigate whether LIF activates calcium dependent signaling molecules, such as calcineurin and calmodulin kinases II and IV  **CaMKII**  and  **CaMKIV** , and, if so, whether these calcium mediated signaling events contribute to LIF mediated cardiac hypertrophy.  Ref: 11073891 Circ Res, 2000
- Transfection experiments using expression plasmids for constitutively active forms of CaMKI,  **CaMKII** , or  **CaMKIV**  show that  **CaMKII**  is the most effective activator of prolactin promoter expression.  Ref: 9324052 Mol Cell Endocrinol, 1997
- The roles of two multifunctional calcium calmodulin dependent protein kinases,  **CaMKIV**  and  **CaMKII** , were examined in transient transfection studies that utilized either the full length or the constitutively active forms of these kinases.  Ref: 7958915 Genes Dev, 1994
- When Ser142 was mutated to alanine, CREB was activated by  **CaMKII** , as well as by  **CaMKIV** .  Ref: 7958915 Genes Dev, 1994
- Calmodulin dependent protein kinase phosphatase CaMKP dephosphorylates and concomitantly deactivates multifunctional calcium calmodulin dependent protein kinases CaMKs, such as CaMKI,  **CaMKII** , and  **CaMKIV** .  Ref: 11726284 J Biochem (Tokyo), 2001
- This finding together with previous reports regarding localization of CaMKs indicates that CaMKP N dephosphorylates  **CaMKIV**  and nuclear  **CaMKII** .  Ref: 11726284 J Biochem (Tokyo), 2001
- The expression of PKAalpha, PKCalpha and  **CaMKII** ... in periglomerular cells suggests that these three kinases may play a role in TH gene regulation in the olfactory bulb.  Ref: 10944503 Chem Senses, 2000
- The results suggest that  **CaMKII**  is involved in LTP induction, while  **CaMKIV**  and MAPK are rather involved in LTP maintenance.  Ref: 12491766 Nippon Yakurigaku Zasshi, 2002
- The changes in nNOS were accompanied by changes in protein expression of calmodulin kinase II  **CaMKII**  P< ; 0.05 and calmodulin kinase IV  **CaMKIV**  P< ; 0.05.  Ref: 11689164 Brain Res, 2001
- Whereas  **CaMKIV**  was primarily found in granule cells,  **CaMKII**  was present in the glomerular, external plexiform, mitral cell and granule cell layers.  Ref: 10944503 Chem Senses, 2000
- METHODS AND We studied a mouse model of cardiac hypertrophy attributable to transgenic TG overexpression of a constitutively active form of  **CaMKIV**  that also has increased endogenous  **CaMKII**  activity.  Ref: 12208807 Circulation, 2002
- The entry into apoptosis was associated with a progressive caspase 3 dependent cleavage of  **CaMKIV** , but not of  **CaMKII** .  Ref: 11149901 FASEB J, 2001
- MEKK and  **CaMKII** ... increased promoter activity 4.5 and 1.5 fold, respectively, in NB2A cells.  Ref: 12421350 J Neurochem, 2002
- We also found that the profile of  **CaMKIV**  and  **CaMKII**  isoforms in primary cortical glial cultures and BV 2 cells is distinct from that found in neurons.  Ref: 11578781 Neurochem IntNeurochem Int, 2002
- **CaMKII**  or  **CamKIV**  activity suffices to support neuronal survival in the absence of other trophic stimuli.  Ref: 12671991 J Neurosci Res, 2003
- With the target enzymes smooth muscle myosin light chain kinase,  **CaMKIV**  and  **CaMKII** , the rates of calmodulin compaction were one two orders of magnitude lower compared with those of the peptides and in the case of alphaCaMKII, ATP binding and Thr 286 auto phosphorylation were required for calmodulin compaction.  Ref: 12023824 Biochem Soc Trans, 2002
- To further study the role of CaMKs, constitutively active forms of CaMKI CaMKI 295, II  **CaMKII**  290, and IV  **CaMKIV**  313 were transfected with CYP11B2 reporter constructs.  Ref: 12193581 Endocrinology, 2002
- **CaMKII** ,  **CaMKIV** , and calcineurin are critically involved in LIF induced cardiac hypertrophy.  Ref: 11073891 Circ Res, 2000
- To determine which CaMK regulates CYP11B2, vectors containing the complete coding sequences for CaMKI,  **CaMKII** , and  **CaMKIV**  were transfected with the CYP11B2 reporter construct.  Ref: 12193581 Endocrinology, 2002
